# The observational clinical registry (cohort design) of the European Reference Network on Rare Adult Solid Cancers: The protocol for the rare head and neck cancers

**DOI:** 10.1371/journal.pone.0283071

**Published:** 2023-03-16

**Authors:** Annalisa Trama, Lisa Licitra, Stefano Cavalieri, Simone Bonfarnuzzo, Paolo Baili, Antonio Ciarfella, Pablo Parente, Giovanni Almadori, Mohssen Ansarin, Almalina Bacigalupo, Philipp Baumeister, Bertrand Baujat, Paolo Bossi, Elisa Cavalera, Maria Cecilia Cercato, Francois Dieleman, Nicolas Fakhry, Virginia Ferraresi, Francesca Gaino, Danilo Galizia, Jana Halamkova, Elina Halme, Jose Hardillo, Benedikt Hofauer, Emma Kinloch, Lorenzo Livi, Laura Deborah Locati, Stefan Mattheis, Giuseppe Mercante, Aurora Mirabile, Gabriele Molteni, Ester Orlandi, Roberto Persio, Stefania Sciallero, Ludi Smeele, Marta Tagliabue, Valentino Valentini, Carla Van Harpen, Christoph Benedikt Westphalen, Laura Botta

**Affiliations:** 1 Department of Epidemiology and Data Science, Evaluative Epidemiology Unit, Fondazione IRCCS Istituto Nazionale dei Tumori, Milan, Italy; 2 Head and Neck Medical Oncology Department, Fondazione IRCCS Istituto Nazionale dei Tumori di Milano, Milano, Italy; 3 Department of Oncology and Hemato-Oncology, University of Milan, Milan, Italy; 4 Department of Epidemiology and Data Science, Data Science Unit, Fondazione IRCCS Istituto Nazionale dei Tumori, Milan, Italy; 5 Department of Otorhinolaryngologyand Head and Neck Surgery, La Corunna University Hospital, Corunna, Spain; 6 Department of Head-Neck Oncology, Fondazione Policlinico Universitario Agostino Gemelli IRCCS, Largo A. Gemelli, Rome, Italy; 7 Head and Neck Program and Division of Otolaryngology Head &Nech Surgery, European Institue of Oncology, Milan, Italy; 8 Radioterapia Oncologica, IRCCS-AOU Ospedale Policlinico San Martino – IST, Genoa, Italy; 9 Department of Otorhinolaryngology, Head and Neck Surgery, University Hospital, Ludwig-Maximilians University, Munich, Germany; 10 Otorhinolaryngology – Head and Neck surgery Department, APHP/Sorbonne Université, Hospital Tenon, Paris, France; 11 Medical Oncology Unit, Department of Medical and Surgical Specialities, Radiological Sciences and Public Health, University of Brescia, ASST Ospedali Civili, Brescia, Italy; 12 Department Oncological Radiotherapy, "Vito Fazzi" Hospital, Lecce, Italy; 13 Epidemiology and Cancer Registry Unit – IRCCS Regina Elena National Cancer Institute, Rome, Italy; 14 Department of Head and Neck Surgical Oncology, UMC Utrecht Centre of Expertise for Head and Neck Cancer, UMC Utrecht Cancer Centre, Utrecht, the Netherlands; 15 Department of Oto-Rhino-Laryngology Head and Neck Surgery, La Conception University Hospital, AP-HM, Aix Marseille University, Marseilles, France; 16 Sarcoma and Rare Tumours Departmental Unit-IRCCS Regina Elena National Cancer Institute-Rome, Rome, Italy; 17 Department of Biomedical Sciences, Humanitas University, Pieve Emanuele (MI), Italy; 18 Department of Otorhinolaryngology - Head and Neck Surgery, IRCCS Humanitas Research Hospital, Rozzano (MI), Italy; 19 Istituto di Candiolo, FPO - IRCCS, Candiolo (TO), Italy; 20 Department of Comprehensive Cancer Care, Masaryk Memorial Cancer Institute, Brno, Czech Republic; 21 Faculty of Medicine, Department of Comprehensive Cancer Care, Masaryk University, Brno, Czech Republic; 22 Department of Otorhinolaryngology - Head and Neck Surgery, Tampere University Hospital, Tampere, Finland; 23 Department of otorhinolaryngology - Head and neck surgery, Erasmus University Medical Centre Rotterdam, Rotterdam, the Netherlands; 24 Comprehensive Cancer Centre Munich, Munich, Germany; 25 Salivary Gland Cancer UK, International House, London, United Kingdom; 26 Department of Oncological Radiotherapy, Azienda Ospedaliero-Universitaria Careggi, Florence, Italy; 27 Department of Medical Oncology of Head and Neck Tumours, ICS Maugeri SpA SB - IRCCS, Pavia, Italy; 28 Ear, Nose and Throat Clinic, Essen Univerity Hospital, Essen, Germany; 29 Department of Medical Oncology, IRCCS San Raffaele, Milan, Italy; 30 Otolaryngology Head and Neck Surgery Department, University Hospital, Verona, Italy; 31 Clinical Department, Radiotherapy Unit, National Centre for Oncological Hadrontherapy (Fondazione CNAO), Pavia, Italy; 32 Italian Association of Laryngectomees, Milan, Italy; 33 Medical Oncology Unit 1, IRCCS Ospedale Policlinico San Martino, Genoa, Italy; 34 Department of Head and Neck surgery, Surgical Oncology Division, Netherlands Cancer Institute, Amsterdam, the Netherlands; 35 Department of Otolaryngology Head & Neck Surgery, IEO, European Institute of Oncology IRCCS, Milan, Italy; 36 Department of Biomedical Sciences, University of Sassari, Sassari, Italy; 37 Unit of Surgical Oncology, Maxillo Facial Reconstruction, Policlinico Umberto I, Rome, Italy; 38 Department Medical Oncology, Radboud University Medical Centre, HB Nijmegen (HP 455), Nijmegen, the Netherlands; 39 Comprehensive Cancer Centre Munich & Department of Medicine III, German Cancer Consortium (DKTK), Ludwig Maximilian University of Munich, Munich, Germany; Abu Dhabi University, UNITED ARAB EMIRATES

## Abstract

**Introduction:**

Care for head and neck cancers is complex in particular for the rare ones. Knowledge is limited and histological heterogeneity adds complexity to the rarity. There is a wide consensus that to support clinical research on rare cancer, clinical registries should be developed within networks specializing in rare cancers. In the EU, a unique opportunity is provided by the European Reference Networks (ERN). The ERN EURACAN is dedicated to rare adults solid cancers, here we present the protocol of the EURACAN registry on rare head and neck cancers (ClinicalTrials.gov Identifier: NCT05483374).

**Study design:**

Registry-based cohort study including only people with rare head and neck cancers.

**Objectives:**

1. to help describe the natural history of rare head and neck cancers;
2. to evaluate factors that influence prognosis;
3. to assess treatment effectiveness;
4. to measure indicators of quality of care.

**Methods:**

Settings and participants

It is an hospital based registry established in hospitals with expertise in head and neck cancers. Only adult patients with epithelial tumours of nasopharynx; nasal cavity and paranasal sinuses; salivary gland cancer in large and small salivary glands; and middle ear will be included in the registry. This registry won’t select a sample of patients. Each patient in the facility who meets the above mentioned inclusion criteria will be followed prospectively and longitudinally with follow-up at cancer progression and / or cancer relapse or patient death. It is a secondary use of data which will be collected from the clinical records. The data collected for the registry will not entail further examinations or admissions to the facility and/or additional appointments to those normally provided for the patient follow-up.

Variables

Data will be collected on patient characteristics (eg. patient demographics, lifestyle, medical history, health status); exposure data (eg. disease, procedures, treatments of interest) and outcomes (e.g. survival, progression, progression-free survival, etc.). In addition, data on potential confounders (e.g. comorbidity; functional status etc.) will be also collected.

Statistical methods

The data analyses will include descriptive statistics showing patterns of patients’ and cancers’ variables and indicators describing the quality of care. Multivariable Cox’s proportional hazards model and Hazard ratios (HR) for all-cause or cause specific mortality will be used to determine independent predictors of overall survival, recurrence etc. Variables to include in the multivariable regression model will be selected based on the results of univariable analysis. The role of confounding or effect modifiers will be evaluated using stratified analysis or sensitivity analysis. To assess treatment effectiveness, multivariable models with propensity score adjustment and progression-free survival will be performed. Adequate statistical (eg. marginal structural model) methods will be used if time-varying treatments/confounders and confounding by indication (selective prescribing) will be present.

**Results:**

The registry initiated recruiting in May 2022. The estimated completion date is December 2030 upon agreement on the achievement of all the registry objectives. As of October 2022, the registry is recruiting. There will be a risk of limited representativeness due to the hospital-based nature of the registry and to the fact that hospital contributing to the registry are expert centres for these rare cancers. Clinical Follow-up could also be an issue but active search of the life status of the patients will be guaranteed.

## Introduction

Despite the rarity of each of the 198 identified rare cancers (RC), collectively they represent 24% of all new cancer cases diagnosed in the EU28/yearly [1]. Differences in survival for RC exist across European countries suggesting the existence of inequalities in healthcare [2]. Rare cancers in general get less scientific consideration and financial support than their more common counterparts. The generation of clinical evidence is more difficult due to the difficulties of conducting clinical trials for to the small number of patients and the paucity of accessible data, including data from cancer registries.

Europe is covered by widespread population-based cancer registries (CRs). However, clinically relevant data, e.g. on detection, staging, treatment and treatment effects tend to lack across CRs [3]. Ad hoc observational studies (i.e. high-resolution study) [4] are costly and time consuming, too often lack detailed information and are not able to provide it in real-time. In its Rare Cancer Agenda 2030 (https://www.rarecancerseurope.org/rce-jarc/rare-cancer-agenda-2030) the JARC recommended developing clinical registries on RC within networks specializing in RCs to support clinical research.

In the EU, a unique opportunity is provided by the European Reference Networks (ERNs). The ERNs are virtual networks of selected institutions targeting rare conditions. Three ERNs are dedicated to rare cancers: EuroBloodNet for rare haematological diseases, PaedCan for paediatric cancers and EURACAN for rare adult solid cancers (RAC). EURACAN is focusing on 10 out of the 12 families of rare cancers (corresponding to 75% of all rare cancers), each corresponding to a EURACAN “domain”: G1, sarcomas; G2, rare neoplasms of the female genital organs and placenta; G3, rare genitourinary cancers; G4, neuroendocrine tumours; G5, rare gastrointestinal cancers; G6, endocrine cancers; G7, rare head and neck cancers; G8, rare thoracic cancers; G9, rare skin/eye melanoma; G10, central nervous system tumours.

An EU-supported project Starting an Adult Rare Tumour Registry (STARTER) began on April 1^st^ 2020 to develop the EURACAN registry.

The registry initiated on the rare head and neck cancers including nasal cavity and paranasal sinuses (incidence rate 0.5/100,000), nasopharynx (incidence rate 0.5/100,000), salivary gland (incidence rate 1.5/100,000) and middle ear cancers (incidence rate 0.03/100,000), corresponding to 2,500, 2,500, about 8,000 and about 200 new cases/year in Europe, respectively (http://rarecarenet.istitutotumori.mi.it/analysis.php). Cancer care for head and neck cancers is complex in particular for the rare ones. Knowledge is limited, diseases often need multidisciplinary approach, including surgery, radiation, and systemic treatments. Patients tend to be older, to have comorbidities and less social support [5]. Moreover, while most head and neck cancers are predominantly squamous cell carcinomas, salivary gland tumours include more than 20 distinct histological subtypes. A similar consideration holds for paranasal sinus cancers. Thus, heterogeneity adds complexity to the rarity.

Our hypothesis is that the registry, gathering data on rare head and neck cancers, will support research to increase their understanding. The new evidence will contribute to develop and/or ameliorate clinical practice guidelines, to support multidisciplinary discussion and consultations for patients with rare head and neck cancers and ultimately to improve quality of care across Europe.

## Materials and methods

Registry aims

1. to help describe the natural history of rare head and neck cancers;
2. to evaluate factors that influence prognosis;
3. to assess treatment effectiveness;
4. to measure indicators of quality of care.

Furthermore, the registry aims to collect information, where available, on the storage of biological samples at the premises of the participating healthcare providers (HCPs). This will facilitate future studies on rare head and neck cancers biology.

Design and setting

The registry is designed to prospectively collect clinical data derived from diagnostic tests and treatments performed by the HCP as part of patient management. The data collected for the registry will not entail further examinations or admissions to the HCP and/or additional appointments to those normally provided. In other words, it will be an observational, registry-based cohort study including only people with rare head and neck cancers.

The EURACAN Registry will include EURACAN HCPs (across 7 different EU member States) and will be open to other expert HCPs, not yet in EURACAN but with proven experience in rare head and neck cancers.

### Inclusion criteria

- Patients with epithelial tumours of nasopharynx; nasal cavity and paranasal sinuses; salivary gland cancer in large and small salivary glands; and middle ear (i.e. squamous carcinoma; adenocarcinoma; neuroendocrine; adenosquamous carcinoma, teratocarcinosarcoma, NUT carcinoma, odontogenic tumours) + neuroendocrine and adenocarcinoma in hypopharynx; oropharynx; larynx; oral cavity and lip + odontogenic carcinoma in oral cavity.
- Adult patients (aged ≥18 years).
- Diagnosis performed or verified by the expert centre entering the patient information in the registry.
- Patients entering the HCP at any clinical phase of the disease (diagnosis, treatment of primary cancer, treatment of recurrence, treatment of M+ etc.). The HCP will collect information on the entire course of the disease regardless of when it started to manage the patient. The HCP can decide, based on its resources, the number of patients on whom it can collect data.
- New patients managed by the HCP from 2021 onwards plus patients managed by the HCP, who are actively followed up at the hospital, with year of diagnosis dating back to maximum 2018.

### Sample size

This is an observational clinical registry which implies a long-term data collection lasting until all the registry objectives are met. Only HCPs that treat at least 100 cases a year of all rare head and neck cancers are EURACAN member. As of April 2022, the registry is activating 10 HCPs and, with time, is envisioning to at least duplicate the number of data providers. Considering that we expected 6 centres in Italy, 2 centres in Germany, 1 centre in Czech Republic, 1 centre in Spain, 2 centres in France (400 cases per year)and the whole rare head and neck cases in The Netherlands (300 patients yearly, based on incidence estimations), we expect about 1700 patients with a rare head and neck cancer yearly.

Due to the observational nature of the registry, sample size justification is based on the precision of the estimates presented in terms of width of two-sided 95% Confidence Interval (CI) for a single proportion using the Simple Asymptotic method (in case of categorical variables) and in form of normal distribution for means (in case of continuous variables). Thus, for example, for a categorical endpoint (e.g., proportion) a sample size of 80 patients (e.g. middle ear cases in 4 years) will achieve a maximum width of 95% CI on estimated proportions of 23.4% (i.e. estimated proportion +/- 11.7%). For continuous endpoints, a sample size of 80 patients will achieve a maximum width of 95% CI on estimated means of 0.46*SD (i.e. estimated mean +/- 0.23*SD, where SD = Standard Deviation).

Table 1 reports the precision of the estimates for categorical and continuous endpoint under different sample size.

**Table 1:** Precision of the estimates expressed as 95% Confidence Interval (CI) for categorical and continuous endpoint accordingly to different sample size.

		
	Categorical endpoint		Continuous endpoint	
Target sample size	max 95% CI	Width of 95% CI (%)	max 95% CI	Width of 95% CI
1000	Estimate +/-0.03	0.06	Estimate +/- 0.06*SD	0.12*SD
250	Estimate +/- 0.06	0.12	Estimate +/- 0.12*SD	0.24*SD
80	Estimate +/- 0.11	0.22	Estimate +/- 0.22*SD	0.44*SD
70	Estimate +/- 0.12	0.24	Estimate +/- 0.23*SD	0.46*SD
60	Estimate +/- 0.13	0.26	Estimate +/- 0.25*SD	0.50*SD
50	Estimate +/- 0.14	0.28	Estimate +/- 0.27*SD	0.55*SD

For the analytical questions involving several different outputs and variables, it is not possible to define a summary of the sample size calculation. For this reason ad hoc analysis plans for each research question are envisioned.

### Data collection and storage

The registry will exploit data available from:

- national or regional registries/databases (DBs) dedicated to rare head and neck cancers (i.e. nasopharynx; nasal cavity and paranasal sinuses; salivary gland; and middle ear cancers)
- HCP registries/DBs;
- ad hoc data collection by HCPs.

The registry is federated thus, data are stored by the data provider (i.e. national/regional registries and/or HCP). At the local level, data are pseudonymised.

### Data analyses

The Personal Health Train (PHT) enables data from multiple organizations to be analysed without identifiable data leaving the organization. By keeping data at its source, no copies of datasets are generated and/or shared with third parties. Vantage6 is the open source implementation of the PHT (https://www.vantage6.ai). Vantage6 uses the mathematical principle of “federated learning”, typically applied to horizontally partitioned data (i.e. organizations have data from different patient cohorts, but with similar characteristics/items). Federated learning is based on the mathematical principle of splitting computations into (a) parts at the station (local HCP or registry) and (b) a central part. The stations share sub-computations with the central server only. If federated learning does not work, the data, after quality validation, will be anonymised and sent to the coordination centre (i.e. National Cancer Institute of Milan [Fondazione IRCCS Istituto Nazionale dei Tumori, Milan-INT]).

Statistical analyses will be performed based on a study protocol (please refer to the Governance section). Queries will be developed, in collaboration with clinical experts, to interrogate the EURACAN rare head and neck cancers registry to generate the descriptive statistics and relevant information needed to plan the analyses envisaged by the study protocol. Queries may include general description of the characteristics of the patients or of the cancer included in the registry such as: gender, mean age, cancer site, cancer histology, stage, treatment at diagnosis etc. Due to the nature of the registry, supplementary statistical analysis plans are envisioned to reply to specific research questions arising over time. Secondary analyses, developed after making observations, expected or unexpected, on the registry will be identified and highlighted as hypothesis generating. Positive finding could be used for developing a rationale for further studies or grants. Several analyses will be performed, but here we report a general data analysis plan based on the four objectives of the registry.

### Data analyses plan

The data analyses will include descriptive statistics showing frequency and patterns of patients’ and cancers’ variables; analytical analyses investigating the association of patients/disease and/or treatment characteristics and health outcomes.

Descriptive statistics will be used to reconstruct the natural history of rare head and neck cancers (e.g. primary tumour growth rate and pattern, its metastatic dissemination, growth of metastases, association with other diseases etc.) and to report indicators about quality of care.

Multivariable Cox’s proportional hazards model and Hazard ratios (HR) for all-cause or cause specific mortality will be used to determine independent predictors of overall survival, recurrence and second primary cancer. Variables to include in the multivariable regression model will be selected based on the results of univariable analysis or using Information Criteria, such as Akaike Information Criterion. According to the specific research question the role of potential confounders and effect modifiers will be evaluated. Confounders will be included in the model and sensitivity analysis will be performed. Stratified analyses will be performed in the presence of effect modifiers that will be detected by testing the interaction between the predictors and the variable.

To assess treatment effectiveness multivariable models with propensity score adjustment that can remove bias due to real world nature of the registry and progression-free survival will be performed.

High proportion of missing data threaten the validity of the inferences/prognostic models. Thus, a maximum of 10% of missing data will be allowed and missing data will be imputed using strategy such as unconditional/conditional mean or expectation maximum.

### Outcomes

Primary outcomes with a time frame of 1 year include:

- Overall survival (all cause and/or cause specific)
- Disease free survival
- Treatment response: the percentage of patients whose cancer shrinks or disappears after treatment. Treatment response expressed as a complete response; partial response; stable disease; progression based on clinical judgment on imaging.
- Cancer treatment observed adverse events: incidence of cancer treatment adverse events as assessed by Common Terminology Criteria for Adverse Events (CTCAE)
- Surgical complications: percentage of patients with surgical complications as assessed by the Clavien-Dindo Classification
- Quality of care: percentage of patients treated according to clinical practice guidelines for head and neck cancer.

Predictors of mortality and/or tumor response include but are not limited to:

- Patients’ Characteristics (age, gender, educational level, tobacco, comorbidity, virus status when appropriate)
- Tumor Characteristics (site, histology, T,N,M, clinical and pathological stage, tumour mutation)
- Treatment Characteristics (radiotherapy alone, surgery alone, systemic therapy alone, multimodal therapy)

Exposures will mainly include different type of treatment (radiotherapy alone, surgery alone, systemic therapy alone, multimodal therapy)

### Data quality checks

Data quality checks aim to assess whether data value are present, valid and believable in terms of:

- Validity: the data should be in the correct format (e.g. allowed only some values, data formats)
- Plausibility: the data should be acceptable (eg, internal consistency; temporal and atemporal)
- Completeness: the data should not have missing values or miss data records

**Fig 1:**
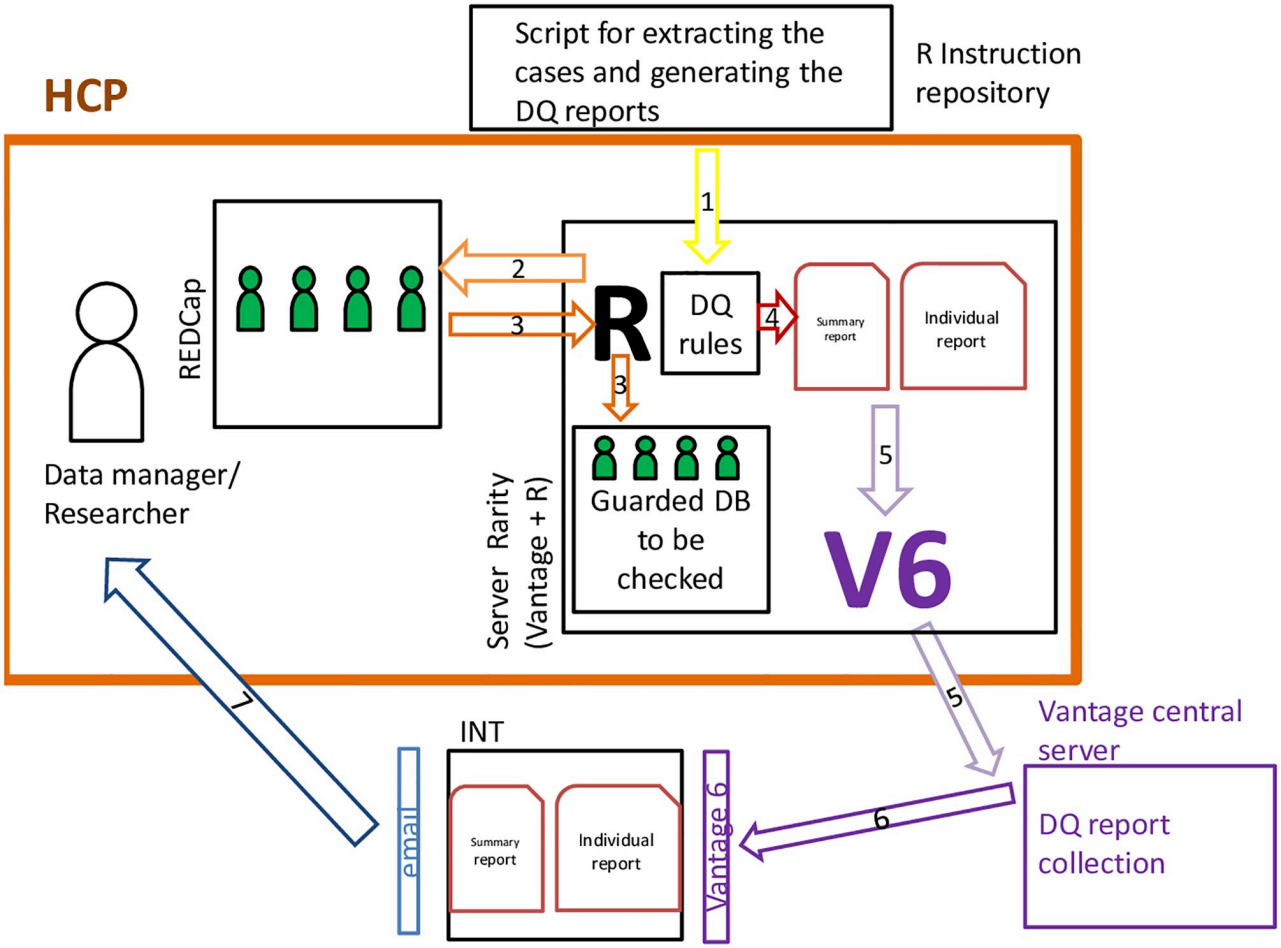
Data quality checks implemented in R.

Validity and plausibility checks are embedded in the electronic case report form (CRF) in the form of alerts and errors during the data input. Additional checks are implemented in R. The R script, including the checks is downloaded locally from an online instruction repository (Fig 1, step 1). The R script extracts from the Research Electronic Data Capture (RedCap) (the IT solution used for the registry CRF) all the completed cases, stores a copy of the DB in the dedicated local server and runs the checks locally (Fig 1, step 2 and 3). The results of these checks are summarized in two reports: a summary and an individual report (Fig 1, step 4). The summary report collects: the number of patients with a complete and valid information on the core data elements; the distribution of treatment by stage for each cancer site included in the registry and the proportion of cases with missing information for each variable. The individual report includes a summary of error for each patient together with a specification of the variables with the errors to be corrected. Thanks to the Vantage6 software, the two reports (not the data) will reach the registry coordination team (INT) to be monitored and discussed with each data provider (Fig 1 step 5, 6 and 7).

After the corrections made by the data providers, all checks will be re-run and quality reports reviewed by INT. Interaction with data providers will be reiterated until sufficient data quality is achieved. The DB with sufficient data quality will be saved and used for the federated learning analysis. These checks will be performed annually and will ensure high data quality within a federated DB. The degree of data completeness will be summarized and available for the researchers and consumers of the analysis of the registry.

### Data to be collected

Core data elements will include the "Set of common data elements for Rare Disease Registration" developed by the Joint Research Centre (JRC) (https://eu-rd-platform.jrc.ec.europa.eu/set-of-common-data-elements) to address specificities of rare cancers as compared to rare diseases.

1. Following the EURACAN registry objectives, data will be prospectively collected on patient characteristics, exposure and outcomes. Patient characteristics are descriptive patient data, such as patient demographics, including lifestyle, medical history, health status, etc. The registry will not collect genetic data. Exposure data focus on the disease, devices, procedures, treatments or services of interest. Outcome data describe patient outcomes (e.g. survival, progression, progression-free survival, death, etc.). In addition, data on potential confounders (e.g. comorbidity; functional status etc.) will be also collected (details in the registry code book on the registry site) [6]. Clinical experts and patients representatives in the global head and neck cancer community were involved in the definition of the included variables. Even if it is impossible to envision all the possible counfounding factors in planning a registry, the community deeply discussed each variable and defined the necessary data to be collected.

### Patient and public involvement

EURACAN has defined European-Patient Advocacy Group (e-PAGs) for each of its domain. G7 e-PAGs were involved in the definition of the registry objectives and of the variables to be collected. They were invited to all the G7 meetings dedicated to the registry since the beginning. Furthermore, an e-PAG Working Group (WG) dedicated to the registry was established to discuss the registry structure, its governance and consent form. G7 e-PAGs representatives were included in this WG. e-PAGs are included in the Registry Steering Committee (please refer to the registry governance section).

### Ethical considerations

The registry was approved by the ethic committee of the Fondazione IRCCS Istituto Nazionale dei Tumori (INT) with protocol number (INT 43/21).

INT is the coordinator of the EURACAN registry as well as a data provider. At the INT, and at each HCP involved, responsible investigators ensure that the EURACAN registry will be implemented in compliance with the protocol, following the instructions and procedures described herein. Each HCP is a controller and will identify a data processor.

Patient protection

Personal data will be recorded and stored in pseudonymised format in a DB installed at the HCP level. Either the PHT solution will enable data sharing among multiple organizations without identifiable data leaving the HCP, or anonymised data will be sent to the INT.

All parties involved in registry development will maintain strict confidentiality to ensure that neither personal privacy nor the privacy of the families of patients participating in the registries will be violated. Data will be processed exclusively by authorized personnel participating in the EURACAN registry (a data processor will be identified at each HCP involved). Access to computer systems and the premises where they are kept will be controlled by appropriate security measures which comply with privacy regulation requirements. The processing of patients’ personal data taking part in the EURACAN registry, and specifically in relation to consent-related data, will comply with local privacy legislation and the General Data Protection Regulation 2016/679 (GDPR) of the European Union.

The registry protocol is submitted to the ethics committees (EC) of the HCPs involved. Furthermore, the ECs of the HCP involved will authorize in advance any research carried out using EURACAN registry data.

Informed consent

Written informed consent is the legal basis of the EURACAN registry and so is required from all participants.

### Status and timeline of the registry

The registry initiated recruiting in May 2022. The estimated completion date is December 2030 upon agreement on the achievement of all the registry objectives. As of August 2022, the registry is recruiting.

## Discussion

Limitations

There is a risk of limited representativeness due to the hospital-based nature of the registry and to the fact that hospital contributing to the registry are expert centres for these rare cancers. Representativeness of the registry will be tested comparing the registry data with population-based data in terms of relevant variables (eg. age, stage, prognosis). Adequate statistical (eg. marginal structural model) methods will be used if time-varying treatments/confounders and confounding by indication (selective prescribing) will be present, not to raise methodological problems.

Directed acyclic graphs can also be useful to identify the source of bias and will be utilized in the definitions of the path between covariates. Close collaboration with clinicians will be crucial also in this part to allow a proper interpretation of the registry data [7]. Clinical Follow-up could be an issue but active search of the life status of the patients will be guaranteed.

Dissemination plan

In order to ensure the appropriate access, use and dissemination of the data, the registry will be supervised by a Steering Committee (SC) with the following tasks:

- to launch, plan, supervise and approve studies and publications based on the registry data
- to ensure adherence to the publication policy and to the guidelines for accessing the registry data
- to plan and endorse modifications of the registry structure (e.g., pathologic classification or staging changes etc.)
- deliberate on requests of enrollment into the EURACAN Registry from non EURACAN centres or networks linked to it
- to discuss and agree amendments to the registry
- once the registry is fully functioning, to promote the use of the registry data also for international collaborative studies (e.g. with US, Rare Cancers Asia, etc.), in other words ensure that the EURACAN Registry will be **FAIR** (findable, accessible, interoperable, reusable)
- to review the EURACAN registry governance
- to identify opportunities for financial support to maintain the registry.

Data access rules

1. data remain the property of the contributing HCPs or registries
2. each HCP or national registry is free to access and use its own data for research purposes
3. each HCP or national registry including data in the EURACAN Registry can request access to the EURACAN Registry federated database upon the presentation of a study protocol that has to be approved by the SC
4. third parties (e.g., pharmaceutical companies, patient organisations, competent authorities etc.) can request use of the registry data. However, data should be used for projects that include EURACAN members. Thus, third parties proposing a research question, should work with a EURACAN Principal Investigator (PI) and should present a study protocol to be reviewed by the SC. If the research question is relevant but does not require a study protocol, a quick review will be provided by the SC and a written report including results of data analyses will be shared with the third party
5. Commercial companies, depending on the study, may be asked to contribute funding. Data do not move from the HCPs or national registries and the commercial companies will not access the EURACAN registry federated database. Funding will be used to support the registry maintenance and/or specific studies proposed by the EURACAN members and based on the registry data
6. each EURACAN domain will define a domain registry working group (RWG), made up of 3 to 5 members including an ePAG, to review the study protocols presented for the domain. Additional expertise may be brought in as required. The domain leader will chair this RWG and will report to the SC the results of the revision to inform and get SC approval
7. once a study on a specific domain is approved by the SC, the scientific secretariat will inform the relevant HCP or national registry by email requesting to express its willingness to share its data for the approved study. If an HCP or national registry does not wish to share its data for the approved study, it can opt out from the study. In case, it is expected that it will provide explicit reason. The HCP or national registry should inform the scientific secretariat within 2 weeks. In the event the HCP or national registry does not confirm whether it wishes to share its data within 2 weeks from the scientific secretariat request, its data will not be used for the study
8. the scientific secretariat will contact the national registry coordinator, not each centre contributing to the national registry (e.g. NETSARC, etc.). This is in line with the vision of EURACAN being a network of networks and therefore also a registry network
9. the scientific secretariat will inform the principal investigator (PI) of the proposed study about the SC decision
10. the PI of the proposed study will be responsible for arranging a preliminary ethical review that will be shared with the participating centres. The participating centres should provide their institutional ethical review response to the PI within 60 days.

The engagement of the EURACAN Registry in international collaborative efforts is highly supported. In this context, the SC is asked to deliberate on collaborative projects involving the registry. Also in this case HCPs and national registries will be informed and will be asked to agree or to opt-out of such engagement.
